# Recent Advances in Anti‐Atherosclerosis and Potential Therapeutic Targets for Nanomaterial‐Derived Drug Formulations

**DOI:** 10.1002/advs.202302918

**Published:** 2023-09-12

**Authors:** Zhicheng Xiao, Yi Li, Liyan Xiong, Jun Liao, Yijun Gao, Yunchun Luo, Yun Wang, Ting Chen, Dahai Yu, Tingfang Wang, Chuan Zhang, Zhe‐Sheng Chen

**Affiliations:** ^1^ Shanghai Engineering Research Center of Organ Repair School of Medicine Shanghai University Shanghai 200444 China; ^2^ Weihai Medical Area 970 Hospital of Joint Logistic Support Force of PLA Weihai 264200 China; ^3^ Department of Pharmaceutical Sciences College of Pharmacy and Health Sciences St. John's University New York 11439 USA

**Keywords:** atherosclerosis, drug, nanodelivery, nanoparticles, therapeutic strategy

## Abstract

Atherosclerosis, the leading cause of death worldwide, is responsible for ≈17.6 million deaths globally each year. Most therapeutic drugs for atherosclerosis have low delivery efficiencies and significant side effects, and this has hampered the development of effective treatment strategies. Diversified nanomaterials can improve drug properties and are considered to be key for the development of improved treatment strategies for atherosclerosis. The pathological mechanisms underlying atherosclerosis is summarized, rationally designed nanoparticle‐mediated therapeutic strategies, and potential future therapeutic targets for nanodelivery. The content of this study reveals the potential and challenges of nanoparticle use for the treatment of atherosclerosis and highlights new effective design ideas.

## Graphical abstract

Atherosclerosis, the leading cause of death worldwide, is responsible for ≈17.6 million deaths globally each year. Diversified nanomaterials can improve drug properties and are considered to be key for the development of improved treatment strategies for atherosclerosis. The pathological mechanisms underlying atherosclerosis is summarized, rationally designed nanoparticle‐mediated therapeutic strategies, and potential future therapeutic targets for nanodelivery.

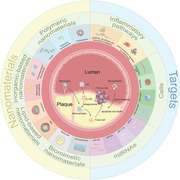

## 1 Introduction

Atherosclerosis (AS) is a complex immunoinflammatory disease involving the release of abnormal proinflammatory cytokines and the accumulation of lipid‐rich plaques, which disturb vascular homeostasis, followed by a series of complications such as coronary artery disease, myocardial infarction, and stroke.^[^ ^1^, ^2^ ^]^ According to the National Institutes of Health, the annual global death toll due to atherosclerosis is ≈17.6 million, making it the leading cause of death worldwide.^[^ ^3^ ^]^ The atherosclerotic pathological environment is characterized by inflammation and the buildup of lipid‐rich plaques in medium to large arteries. This limits the potential of AS theragnostic therapies. Currently, antiplatelet, hypolipidemic, and vasodilating drugs are primarily used in the clinical treatment of atherosclerosis.^[^ ^4^ ^]^ Pharmacological therapies can efficiently control the progression of atherosclerosis; however, to date, the use of oral medications has not had satisfactory therapeutic effects owing to factors such as poor bioavailability, nonspecific distribution, and high toxicity.^[^ ^5^ ^]^ For instance, the Food and Drug Administration (FDA) has approved the antioxidant Probucol, a typical drug, to treat atherosclerosis by controlling lipids and antioxidants.^[^ ^6^ ^]^ However, probucol has a low oral bioavailability of < 10% and it does not easily enter into systemic circulation via the gastrointestinal tract.^[^ ^7^ ^]^ Most of this drug is consequently excreted in its original form. Its clinical applicability is thus very constrained, and its efficacy is difficult to fully exploit. To overcome the treatment bottleneck for atherosclerosis, it is crucial to identify efficient treatment strategies and medications.

Nanomaterials have provided unprecedented opportunities to overcome these challenges. Specifically, extensive studies have demonstrated that nanocarriers can improve a drug's physicochemical properties, enabling it to evade the phagocyte system and reach the treatment area, via modifications such as inclusion of targeted ligands, the use of biomimetic materials, and the use of materials responsive to a certain microenvironment.^[^ ^8^, ^9^, ^10^ ^]^ Additionally, several studies have demonstrated that nanotechnologies can enhance drug availability and blood concentration and, thus, the therapeutic impacts for various diseases, including AS.^[^ ^11^, ^12^ ^]^ Some nanomaterials, such as CeO_2_ and cyclodextrin, which have reactive oxygen species (ROS) scavenging and lipid clearance capabilities, respectively, have beneficial effects on AS therapies due to their inherent structural characteristics.^[^ ^13^, ^14^, ^15^ ^]^ More importantly, AS has been treated with a range of nanoparticles (NPs), including those that promote cholesterol efflorescence, are anti‐inflammatory, improve oxidative stress, and inhibit senescence.

**Figure 1:**
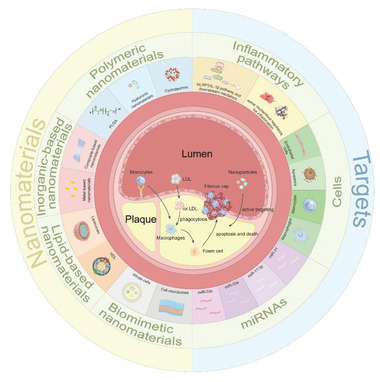
A schematic overview of the nanomaterials used to treat microenvironments containing vulnerable atherosclerotic plaques.

The use of nanomaterials in the treatment of atherosclerotic plaques has been summarized in the literature. However, to the best of our knowledge, a thorough and systematic review on the targeting strategies using nanomaterials in the treatment of vulnerable atherosclerotic plaques, which considers the plaque microenvironment, has not yet been published. Therefore, the purpose of this review is to provide a comprehensive overview of recent advances in nanotherapies that have been designed specifically AS theranostics. First, an essential background is provided to summarize the mechanisms of AS and potential therapeutic targets, and this is followed by a summary and analysis of three commonly used nanomaterials in the development of therapies against AS, as illustrated in **Figure** **1**. Finally, a summary of nanotherapy‐based approaches used to treat atherosclerotic lesions is provided, to reveal the potential and challenges of this strategy.

## 2 Mechanisms and Pathological Features of AS

Atherosclerosis, the dominant pathological basis for the occurrence and development of cardiovascular diseases, is a chronic inflammatory disease affecting large and medium‐sized arteries, and it results in plaque formation. The pathological progression of AS can be divided into four stages: early fatty streaks, fibrous plaques, atherosclerotic plaques, and complicated lesions or secondary changes.^[^ ^16^ ^]^

Endothelial cells (ECs), which are critical components of the blood vessel wall, are connected by tight junctions that separate the bloodstream from the surrounding vascular wall tissue. The early stages of AS are defined by EC dysfunction occurs. This is principally induced by the consumption of an atherogenic diet, leading to endothelial nitric oxide synthase (NOS) dysfunction and secondary reduction of the levels of nitric oxide (NO).^[^ ^17^, ^18^ ^]^ NO has various vascular protective functions, such as anti‐inflammatory effects, inhibition of leukocyte chemotaxis, and regulation of vascular dilation.^[^ ^19^, ^20^ ^]^ Therefore, its decrease can promote vascular inflammation leading to the activation of ECs, thus mediating enhanced monocyte recruitment and inflammatory cascade reactions.^[^ ^21^ ^]^

After being recruited into the intimal space, monocytes differentiate into macrophages in response to locally produced macrophage colony‐stimulating factor (M‐CSF) and other cytokines and, depending on the different signals in their local microenvironments, they acquire corresponding phenotypes [proinflammatory (M1) and anti‐inflammatory (M2)].^[^ ^22^, ^23^ ^]^ Proinflammatory macrophages are characterized by the production of high levels of proinflammatory cytokine, ROS, and reactive nitrogen species.^[^ ^24^ ^]^ Inflammatory macrophages ultimately ingest oxidized low density lipoprotein (LDL) through scavenger receptors to become foam cells, thereby promoting plaque formation.^[^ ^2^ ^]^ Although foam cells can use the transporters ATP‐binding cassette subfamily A member 1 and subfamily G member 1 to expel excess cholesterol and transport it to extracellular apolipoprotein A‐I (apoA‐I) and high‐density lipoprotein (HDL), they often undergo apoptosis or necrosis, producing a growing “necrotic core” composed of cholesteryl esters, cellular debris, and cholesterol crystals, increasing the likelihood of a lesion area rupture.^[^ ^25^, ^26^, ^27^ ^]^

In addition, during lesion growth, smooth muscle cells (SMCs) change from a contracted state to a proliferative state, and then migrate into the subendothelial space to create a “fibrous cap” that protects the lesion areas from rupture. The thinning of the fibrous cap and the enlargement of the necrotic core can lead to plaque rupture, which exposes prothrombotic substances within the plaque, triggers platelet activation, and leads to arterial occlusion.^[^ ^28^ ^]^

## 3 Potential Inflammation‐Related Signaling Pathway and Cell Targets for the Treatment of AS

A low‐fat diet and medications to lower cholesterol levels are currently the most frequently used treatments for AS; however, clinical outcomes from their use have not been ideal. The main factor limiting the advancement of current anti‐AS drugs is their lack of specificity. Altered cholesterol metabolism, oxidative stress, hypoxia, apoptosis, and cell necrosis are known to have significant roles in the development of AS within its immunological microenvironment. Thus, reshaping the atherosclerotic microenvironment can reduce AS. This section discusses prospective therapeutic targets related to the different factors defining the atherosclerotic lesion microenvironment.

### 3.1 Inflammatory Pathways

#### 3.1.1 NOD‐, LRR‐, and Pyrin Domain‐Containing protein 3 (NLRP3)/Interleukin (IL)−1β Signaling Pathway and its Downstream Mediators

AS is a chronic inflammatory disorder whose initiation and progression are influenced by various inflammatory mediators.^[^ ^29^, ^30^ ^]^ Identifying targets to effectively reduce inflammation could aid in the development of new techniques to lower the incidence of cardiovascular events and the risks associated with atherosclerotic vessel walls.

Aberrant inflammasome activation and the release of IL‐1β accelerates AS lesion development in mice. These lesions can be greatly reduced by the pharmacological inhibition of NLRP3 and IL‐1β.^[^ ^31^ ^]^ Tumurkhuu et al. showed that NLRP3 inflammasome‐induced IL‐1β suppresses ATP‐binding cassette transporter A1 (ABCA1)‐mediated cholesterol efflux by downregulating the Gpr109a receptor, which controls ABCA1 expression.^[^ ^32^ ^]^ IL‐1 is composed of two primary cytokines, IL‐1α and IL‐1β; the latter being a major proinflammatory mediator of AS.^[^ ^33^ ^]^ The NLRP3 inflammasome serves as a platform for pro‐caspase‐1 activation to caspase‐1^[^ ^34^ ^]^ and thereby, facilitates the processing of its substrates, including pro‐IL‐1β. Subsequently, mature bioactive IL‐1β is released.^[^ ^35^ ^]^ In the early stages of systemic inflammation, IL‐1β is the proinflammatory cytokine that triggers the production of several other inflammatory mediators such as IL‐6 and tumor necrosis factor‐α (TNF‐α).^[^ ^36^ ^]^ Therefore, by targeting the NLRP3 inflammasome, IL‐1β, and its downstream inflammatory mediators (IL‐6 and TNF‐α), the progression of AS can be inhibited. There are three main strategies that can be used to help prevent AS from progressing: 1) decreasing the level of IL‐1β by repressing the dynamic NLRP3 inflammasome. This therapeutic strategy targeting the inflammasome signaling pathway represents an important step toward clinical application. The CANTOS trial confirmed that inflammasome activation is a cause rather than a consequence of atherosclerotic thrombosis.^[^ ^37^ ^]^ As drugs targeting IL‐1 enter clinical trials, researchers are faced with new challenges and opportunities. 2) Inhibiting IL‐1 receptors to restrict or cancel the activity of IL‐1β; and 3) suppressing the activity of secondary proinflammatory factors (IL‐6, TNF‐α) to decrease the inflammatory responses in cells. For example, ziltivekimab is a fully human monoclonal antibody that reduces inflammation throughout the body by inhibiting IL‐6. Results from the randomized, double‐blind, placebo‐controlled phase II trial RESCUE indicated that ziltivekimab significantly reduced multiple inflammatory biomarkers associated with AS.^[^ ^38^ ^]^

#### 3.1.2 Molecular Regulators of Inflammation

##### Proprotein Convertase Subtilisin/Kexin type 9 (PCSK9)

PCSK9 is a member of the pro‐protein convertase family and it supports atherogenic functions autonomously via its regulatory effects on plasma lipid levels.^[^ ^39^ ^]^ Low‐density lipoprotein (LDL) catabolism is inhibited, and plasma levels of LDL‐cholesterol are increased as circulating PCSK9 binds to the LDL receptor (LDLR) and directs it toward lysosomal degradation within cells.^[^ ^40^ ^]^ As a result, efforts have been made to boost LDLR expression and lower plasma LDL‐C levels using PCSK9 as a therapeutic target. Strategies for inhibiting PCSK9 include gene silencing and the use of mimetic peptides and monoclonal antibodies.^[^ ^41^, ^42^ ^]^

In 2015, the FDA approved the clinical use of two fully humanized monoclonal antibodies: evolocumab (Repatha from Amgen) and alirocumab (Praluent from Regeneron and Sanofi). Since then, PCSK9 monoclonal antibodies have shown a flourishing trend, and now include 1B20 (Merck), bococizumab (Pfizer), JS002 (Junshi Bio), and frovocimab/LY3015014 (Eli Lilly). Among them, the bococizumab and frovocimab antibodies have been shown to lower LDL‐C levels in the same manner as alirocumab and evolocumab.^[^ ^43^, ^44^ ^]^ While, inclisiran (Leqvio from Alnylam and Novartis), a class of siRNA‐based inhibitor, was found to target PCSK9 mRNA degradation and lower PCSK9 levels. Inclisiran was given FDA approval in December 2021 for subcutaneous injection cholesterol reduction.^[^ ^45^ ^]^ Even more exciting is that MK‐0616 (Merck), an oral macrocyclic peptide inhibitor for the treatment of hypercholesterolemia, was shown to safely and effectively reduce plasma LDL cholesterol levels in patients with hypercholesterolemia in a dose‐dependent manner in Phase II clinical trials.^[^ ^46^ ^]^ In conclusion, FDA‐approved antibodies that target PCSK9 have already demonstrated clinically effective atherosclerotic cardiovascular disease risk reduction and LDL‐C lowering without the presence of significant adverse effects. Additionally, several alternative methods for targeting PCSK9 are in development, and a siRNA‐based technique targeting PCSK9 has received FDA approval. Finally, novel PCSK9 inhibitor applications are also being developed, which will further increase the potential of PCSK9 targeting.

##### Protease‐Activated Receptors

Cell signaling in atherosclerotic plaques can be mediated by protease‐activated receptors (PARs), and to date, four isoforms have been discovered (PAR1‐4).^[^ ^47^ ^]^ Thrombin and activated factor X are two proteases that can activate PAR both systemically and at lesion sites. After activating PAR and their downstream signaling, these proteases can cause various effects, including proinflammatory effects and hypercoagulability,^[^ ^48^ ^]^ depending on the protease type, activated PAR, and location of the activation. Proinflammatory reactions are primarily observed following PAR1 and PAR2 activation, whereas hypercoagulability is primarily mediated by PAR1 and PAR4.^[^ ^49^ ^]^ Based on this pathophysiological role and their contributions to AS, PAR signaling pathways could be inhibited to help avoid cardiovascular events.

### 3.2 Cellular Therapeutic Targets

#### 3.2.1 ECs

Key events in the initial stages of AS include endothelial dysfunction and the inflammatory stimulation of ECs.^[^ ^50^ ^]^ Through the production of mediators such as tissue plasminogen activator, prostacyclin, NO, and antithrombin III, ECs serve as the guardians of vascular homeostasis, as they prevent blood clotting, platelet activation, and leukocyte adherence and infiltration.^[^ ^51^ ^]^ The equilibrium between vasodilation and vasoconstriction is disturbed when ECs are damaged, resulting in events that can worsen AS. When considering activated ECs in atheromatous plaques, available active targets may include expressed adhesion molecules such as E‐selectins, vascular cell adhesion molecule 1 (VCAM‐1), and intercellular adhesion molecule 1 (ICAM‐1).^[^ ^52^ ^]^

#### 3.2.2 Monocytes

Initiating adaptive immunity and regulating innate immune responses are both largely mediated by circulating, dynamic monocyte populations, which are easier to target therapeutically than tissue‐resident immune cells.^[^ ^53^ ^]^ The chemokine/chemokine receptor pair monocyte chemotactic protein 1 (MCP‐1)/ C‐C chemokine receptor‐2 is necessary for the vascular recruitment of inflammatory monocytes.^[^ ^54^ ^]^ Furthermore, monocytes secrete MCP‐1, whose level of expression is high in atherosclerotic plaques.^[^ ^55^ ^]^ The development of therapeutic strategies for inhibiting MCP‐1 secretion, thus inhibiting monocyte chemotaxis, could be a highly effective therapeutic approach for AS. Yin et al. prepared yeast‐derived microcapsules for the delivery of bindarit, a specific synthetic inhibitor of MCP‐1, for the treatment of AS.^[^ ^56^ ^]^ In addition, Leuschner et al. suppressed highly expressed CCR2 in inflammatory cells using liposomal‐loaded siRNA, while germ‐line deletion of CCR2 or MCP‐1 ameliorates illness.^[^ ^57^ ^]^ The number of monocytes and macrophages at the location of atherosclerotic plaques drastically decreased after three weeks of injection of *ApoE^−/−^* mice, and the size of aortic root lesions was significantly diminished.

#### 3.2.3 Macrophages

Most tissues in the body contain tissue‐resident macrophages, and the diversity of the tissue environments in which macrophages reside leads to phenotypic and functional heterogeneity.^[^ ^58^ ^]^ Classically activated M1 macrophages are activated by T helper type 1‐derived cytokines such as interferon (IFN)‐γ and lipopolysaccharide.^[^ ^59^ ^]^ They produce proinflammatory cytokines such as IL‐12, IL‐23, and TNF‐α as well as chemokines (CCL5, CXCL9, CXCL10, and CXCL5), which exacerbate the inflammatory state.^[^ ^60^ ^]^ Macrophages have emerged as key targets in AS therapies because of their crucial role in the development and remission of atherosclerotic lesions.

#### 3.2.4 Apoptotic Cells

Apoptosis is a kind of programmed cell death, and apoptotic cells in the body are mainly removed by phagocytes, predominantly macrophages, via efferocytosis.^[^ ^61^ ^]^ Complete and effective efferocytosis can remove apoptotic cells from the body in a timely manner, which is important for maintaining the homeostasis of the body under normal physiological conditions and restoring it under disease conditions.^[^ ^62^ ^]^ Although programmed cell death increases during atherosclerosis, apoptotic cells in the early stages of atherosclerosis can be efficiently removed by the efferocytosis system.^[^ ^63^, ^64^ ^]^ However, as the disease progresses, this balance is disturbed and diseased macrophages are unable to effectively remove lesions and apoptotic cells from the atherosclerotic plaques. Although the mechanisms driving this pathology remain an area of active research, new data suggest that changes in the expression of signaling molecules associated with efferocytosis is the predominant mechanism leading to efferocytosis dysfunction.^[^ ^65^ ^]^ For example, Prakash et al. determined a mechanistic function for myeloid‐specific pyruvate kinase muscle 2 in controlling macrophage inflammation, efferocytosis, most likely via LRP‐1, and hence atherosclerosis. Targeting nuclear PKM2 may thus be a viable strategy by which to slow the growth of atherosclerotic lesions.^[^ ^66^ ^]^ The recent discovery of the role of efferocytosis in vascular disease has significantly deepened our understanding of the mechanisms underlying atherosclerosis and the reasons that necrotic debris builds up over time. If new proefferocytic medicines that allow for the clearance of sick and apoptotic cells are found to be effective and safe in cancer trials, it may be possible to develop a completely new therapeutic platform that directly targets the necrotic core.

### 3.3 MicroRNAs (miRNAs)

miRNAs play a role in the pathophysiology of AS via the regulation of AS‐related genes as well as the post‐transcriptional regulation of gene expression. Thus, by influencing the levels of proteins synthesized within cells, they may play an important role in promoting the dysregulation that affects ECs, white blood cells, and smooth muscle cells, thereby initiating and increasing the growth of atherosclerotic plaques.^[^ ^67^ ^]^ The need for the development of novel methods to combat AS has drawn attention to the means by which we regulate EC inflammation and helped to identify new possible therapeutic targets for AS. These include miR‐31 and miR‐17‐3p that regulate EC activation by directly inhibiting the production of E‐selectin and ICAM‐1.^[^ ^68^ ^]^ In addition, miRNAs are key regulators in cholesterol homeostasis, regulating inflammatory mediators and preventing plaque rupture. For example, miR‐33a and miR‐33b act as post‐transcriptional inhibitors of ABCA1 and ABCG1 expression in macrophages, resulting in a reduced cholesterol efflux to HDL.^[^ ^69^ ^]^ A potential approach to restore normal miRNA expression levels may be through the downregulation of overexpressed or upregulation of repressed miRNAs.

## 4 Therapeutic Nanomaterials for Atherosclerosis

In recent years, NPs have become viable alternative materials for advanced medical diagnostics and therapeutic applications. Three of the most significant nanomaterials currently used in AS‐developing therapy are discussed.

### 4.1 Polymeric‐Based Nanomaterials

Polymeric NPs are commonly employed in nanomedicine research because they are made of biodegradable and biocompatible polymeric materials that are either hydrophobic, hydrophilic, or amphiphilic in nature. Polymeric NPs vary in their size, shape, and drug release qualities and can thus be utilized as drug delivery carriers in a wide range of therapies. Many polymers have been developed to target the sites of atherosclerotic lesions. In this chapter, the use of polymers in the treatment of AS based on the characteristics of the disease is reviewed.

#### 4.1.1 Cyclodextrins

Cyclodextrins are a family of cyclic oligosaccharides consisting of multiple glucopyranose units joined via alpha 1–4 glycosidic bonds.^[^ ^70^ ^]^ As both the oxygen and hydrogen atoms in the glycoside point to the cavity of this circulating structure, it is hydrophobic, whereas cyclodextrin is a water‐soluble complex.^[^ ^71^ ^]^ It should be noted that all cyclodextrin classes have the propensity to aggregate via self‐assembly in aqueous solutions, which decreases their solubility. However, their solubility can be improved by chemical modification.^[^ ^72^ ^]^ For instance, in comparison to unmodified cyclodextrins, modified cyclodextrins such methyl‐cyclodextrin, 2‐hydroxypropyl‐cyclodextrin, and trimethyl‐cyclodextrin are more water soluble, less toxic, and better at forming complexes.^[^ ^73^ ^]^ Cyclodextrin is mainly used as a complexing agent to enhance the water solubility of drugs, reduce their adverse effects, and prolong their circulation time.^[^ ^14^ ^]^ Among them, β‐cyclodextrin has been utilized extensively in the treatment of AS because of its in vivo effectiveness to regulate cholesterol metabolism and its cavity diameter, which is suitable for medicinal molecules.^[^ ^74^ ^]^ Wang et al. used a sequential‐targeting nanoplatform that included β‐cyclodextrin containing discoidal reconstituted HDL to accelerate cholesterol efflux in foam cells.^[^ ^75^ ^]^ The cholesterol efflux proportion increased by 59.82% at 4 h after the administration of the sequential‐targeting nanoplatform, showing that it substantially improved the cholesterol efflux.

#### 4.1.2 Hyaluronic Acid‐Derived Nanomaterials

Hyaluronic acid (HA) is a natural anionic polysaccharide composed of alternating D‐glucuronic acid and N‐acetyl‐D‐glucosamine units.^[^ ^76^ ^]^ HA contains a variety of hydrophilic groups, including hydroxyl, carboxyl, and acetamido groups. These groups can form intracellular hydrogen bonds, resulting in hydrophilicity and high solubility.^[^ ^77^ ^]^ Furthermore, the axial hydrogen atoms (C‐H) in HA form a hydrophobic domain, allowing HA to acquire amphiphilic properties that are useful for the formation of nanoparticles or micelles. Under physiological conditions, HA has a pKa of ≈3–4, and its carboxyl groups dissociate.^[^ ^78^ ^]^ This means that HA is a negatively charged substance that can bind to positively charged drugs through electrostatic interactions. This characteristic can be employed for medication loading or the creation of nanoparticles. For example, Essam et al. coated HA on simvastatin (SIM) in a zeolitic imidazolate framework‐8 (ZIF‐8) (SIM/ZIF‐8) in an aqueous solution via electrostatic interactions.^[^ ^79^ ^]^

The specific binding ability of HA to the cluster of differentiation 44 (CD44) receptor,^[^ ^80^ ^]^ has facilitated the development of several targeted nanoplatforms for the treatment of AS. These are highlighted in Section 5 of this review. Besides its targeting ability, HA can also increase the circulation time of NPs. It is acknowledged that when NPs are injected into blood, they usually form a protein corona,^[^ ^81^ ^]^ which facilitates their clearance by the reticular system and can also suppress their active targeting ability.^[^ ^82^ ^]^ Notably, HA offers an alternative to polyethylene glycol (PEG), which is commonly used, to reduce the protein absorption and immunogenicity of NPs. In an *ApoE^−/−^* mouse model, Sun et al. engineered a nanosystem modified with surface HA to significantly extend the circulating half‐life of curcumin by 6 times and increase the accumulation of the drug in atherosclerotic lesions by 3.5 times after intravenous administration.^[^ ^83^ ^]^

#### 4.1.3 Poly(Lactic‐Co‐Glycolic Acid)

The catalyzed ring‐opening copolymerization of lactic and glycolic acids units results in the formation of Poly(Lactic‐Co‐Glycolic Acid) (PLGA).^[^ ^84^ ^]^ Monomeric units are sequentially joined during polymerization by ester bonds, creating the PLGA copolymer. Owing to its biocompatibility and efficient biodegradability, accomplished by the hydrolysis of lactate and glycolate ester linkages, PLGA is frequently employed in nanomedicine.^[^ ^85^ ^]^ The monomers resulting after PLGA degradation are digested in the Krebs cycle, producing harmless byproducts (H_2_O and CO_2_).^[^ ^86^ ^]^ The good biocompatibility and biodegradability of PLGA has led to its use in the creation of NPs that are capable of carrying a range of medications against AS.^[^ ^87^, ^88^, ^89^ ^]^ Additionally, ligands can be used to alter the surfaces of PLGA‐derived NPs to target drug delivery to certain disease‐affected locations. Simvastatin‐chitosan‐metronidazole‐PLGA nanoparticles were created by Guo et al. and coated with *Porphyromonas gingivalis*‐treated macrophage membranes.^[^ ^90^ ^]^ These NPs reduced the amount of *Porphyromonas gingivalis* and successfully changed the inflammatory M1‐like state of macrophages to the immunosuppressive M2‐like phenotype.

#### 4.1.4 Other Polymeric Nanomaterials

Polymeric micelles are self‐assembled structures composed of amphiphilic polymers. Each consists of a “core,” which is usually the hydrophobic part, and an outer part (also called “shell”) representing the hydrophilic block of the copolymer structure.^[^ ^91^ ^]^ The hydrophobic core of the micelle can capture lipophilic medicines, and the micelles target the lesion area through an improved permeation retention effect, increasing drug bioavailability.^[^ ^92^ ^]^ Polypropylene, polystyrene, and polylactic acid are the most frequently utilized polymers for the construction of hydrophobic cores.^[^ ^93^ ^]^ Most of these hydrophobic elements are inactive carrier substances with no therapeutic potential. In other words, they do not improve the therapeutic efficacy and increase the costs and toxicity risk of the drug delivery method. Consequently, researchers are now investigating nanocarriers that have their own therapeutic effects. For example, Deborah et al. created peptide amphiphile micelle NPs containing MCP‐1 peptide as the targeting moiety, for the delivery of miR‐145 (a regulator of phenotypic changes in vascular smooth muscles) to atherosclerotic lesions.^[^ ^94^ ^]^ The high‐affinity ligand of CCR2, which is highly expressed in synthetic vascular SMCs, is the MCP‐1/C‐C motif chemokine ligand 2 (CCL2). By including miR‐145 molecules in micelles, they were shielded against premature extracellular release and nuclease‐mediated destruction. In both early‐ and mid‐stage murine atherosclerosis models, miR‐145 micelles determined a reduction in the plaque lesion size and necrotic core area, while maintaining the collagen‐containing structures.

Besides the materials mentioned above, various types of natural and synthetic polymers have been used in the development of drugs for the treatment of AS. Natural polymers include chitosan^[^ ^95^ ^]^ and polymer network hydrogels,^[^ ^96^ ^]^ while synthetic polymers include PEG,^[^ ^97^ ^]^ poly lactic acid,^[^ ^98^ ^]^ and poly vinyl alcohol.^[^ ^99^ ^]^ Chitosan is an acid soluble natural excipient with excellent pH sensitivity, biodegradability, biocompatibility, non‐toxicity, non‐immunogenicity, and strong mucosal adhesion and is safe for oral treatment in vivo.^[^ ^100^ ^]^ However, the use of chitosan can be limited by its poor solubility at neutral pH, so it is usually used after modification.^[^ ^101^ ^]^ Wang et al. grafted alginate to N‐succinylated chitosan to form a nanomatrix (NSC), and then added the drug MGF to the nanomatrix to form a PH‐responsive nano‐delivery system for the treatment of atherosclerosis. The MGF‐loaded nanoparticles produced significant hypoglycemic and hypolipidemic responses after oral administration in diabetic rats.^[^ ^102^ ^]^ Poly vinyl alcohol and poly lactic acids are effective biodegradable copolymers that are approved by the Food and Drug Administration for biomedical applications.^[^ ^103^ ^]^ Atanasio Pet al. prepared nanoparticles using functionalized polylactic acid polyethylene glycol loaded bevacizumab and immune uterine globulin‐1. By attaching specific target molecules (such as antibodies, ligands or receptors) to the surface of nanoparticles, the stability and targeting of the nanoparticles is improved.^[^ ^87^ ^]^ Thus, polymers can be utilized in medicine to encapsulate medications for controlled and slow release, and they are a promising strategy by which to improve the efficiency of treatment administration. Future treatments for current problems associated with AS may be greatly aided by the development of novel polymers.

### 4.2 Lipid‐Based Nanomaterials

#### 4.2.1 Liposomes

Liposomes are vesicle bilayers composed of natural phospholipids and cholesterol with an aqueous core.^[^ ^104^ ^]^ They are typically biocompatible and biodegradable, making them appropriate for use in clinical settings.^[^ ^105^, ^106^ ^]^ Since their development in the 1960s, liposomes have shown promising results in the treatment of AS and other cardiovascular diseases.^[^ ^13^ ^]^

Liposomes can be used as cholesterol scavengers to reduce circulating cholesterol levels as they promote cholesterol efflorescence by upregulating the expression of *ABCA1* and *ABCG1* genes.^[^ ^107^ ^]^ In addition, Gong et al. anchored ginsenoside Rb1 in the hydrophobic region of soybean‐derived liposomes to prepare nano‐cavernous liposomes, which markedly improved the lipid bilayer's attraction for free cholesterol and the melting of cholesterol crystals.^[^ ^108^ ^]^

Besides acting as scavengers, liposomes have been employed as drug delivery systems. They can also be utilized to improve drug stability, reduce drug toxicity, and enable targeted drug delivery at atherosclerotic plaque sites.^[^ ^109^ ^]^ To target the endothelium of atherosclerotic plaques or the macrophages recruited to them, the surfaces of liposomes can be altered with biomimetic materials, such as natural ligands, biomimetic membranes, and peptides.^[^ ^110^ ^]^

#### 4.2.2 HDL‐Derived Nanomaterials

HDL‐derived nanomaterials represent natural NPs composed of phospholipids and apoA‐I.^[^ ^111^ ^]^ Many of the intrinsic characteristics of HDL can be utilized to create desirable medication delivery systems. First, HDL, also known as “good cholesterol”, transports extra cholesterol from the peripheral tissues to the liver for catabolism or elimination by reverse cholesterol transfer.^[^ ^112^ ^]^ Second, HDL particles have anti‐infective, anti‐oxidant, anti‐apoptotic, and anti‐inflammatory characteristics.^[^ ^113^ ^]^ Third, being an endogenous molecule, HDL has exceptional biocompatibility and can evade the mononuclear phagocyte system. Additionally, it has innate receptor ligands that contribute to the direct appearance of its biological activities.^[^ ^114^ ^]^

However, HDL‐based delivery methods have numerous drawbacks, including their high costs and insufficient safety levels. Recombinant HDL (rHDL)‐derived NPs, which are innovative therapeutic delivery vehicles for the targeted distribution of medicines or genes for the treatment of atherosclerotic plaques, have been employed to overcome these problems.^[^ ^115^ ^]^ Unfortunately, some types of rHDL maintain the ability of endogenous HDL of interacting with lecithin cholesterol acyltransferase in the bloodstream. This may compromise the delivery of medication to target cells due to drug leakage.^[^ ^116^ ^]^ Functional moieties should be considered to create rHDL nanoplatforms with greater plaque‐targeting capabilities and improved biological functions to address these problems.

### 4.3 Biomimetic‐Based Nanomaterials

There have been numerous studies investigating the use of NPs for the treatment of AS. Conventional nanomaterials generally have several drawbacks, including poor biocompatibility and insufficient targeting.^[^ ^11^ ^]^ New nanoplatforms targeting atherosclerotic plaques are becoming increasingly popular owing to recent advances in bionanotechnology. Bionanomaterials have greater therapeutic efficacy, longer‐lasting blood circulation, and more effective immune evasion than standard NPs.^[^ ^117^ ^]^ Biomimetic NPs can be broadly divided into cells modified with targeting ligands that mimic cell surface proteins, NPs coated with cell membranes, and whole cells.

#### 4.3.1 Whole Cells

Many circulating cell types, including red blood cells (RBCs), macrophages, and monocytes, have high homing abilities and have been used as drug delivery vehicles.^[^ ^118^ ^]^ They are used to organically identify disease locations and thus, fulfill the “hitchhiking” objective. Guo et al. created cyclodextrin‐decorated macrophages by altering the cell membrane with a β‐cyclodextrin derivative (CD‐MP).^[^ ^119^ ^]^ Through interactions between β‐cyclodextrin and adamantane (ADA), ADA‐modified quercetin‐loaded liposomes can be attached to CD‐MP to create a macrophage‐liposome conjugate. In vivo studies demonstrated the targeted delivery of macrophage‐NPs, showing that macrophages worked “hand‐in‐hand” with liposomes and accumulated in the aortic plaques. Macrophage‐Cy5‐NPs‐treated mice displayed a significant red fluorescence signal in the aorta, with an ≈3.9‐fold greater intensity than that of the Cy5‐NPs‐treated group. The results showed that the plaque‐targeted delivery efficiency of NPs was greatly increased by their supramolecular conjugation with macrophages.

#### 4.3.2 NPs Coated with Cell Membranes

Unique components of cell membranes in the complex atherosclerotic microenvironment can lead to highly specific interactions with NPs.^[^ ^120^ ^]^ Therefore, synthetic NPs coated with natural cell membranes are now considered an appealing nanoplatform for theranostic drug delivery. In a recent study, Li et al. created extracellular vesicles (EVs) from mesenchymal stem cells treated with platelets.^[^ ^121^ ^]^ Alteration of the platelet membrane caused the platelets to inherit an innate propensity to adhere to atherosclerotic plaques. Subsequently, an increase in plaque macrophage uptake occurred enhancing the therapeutic efficacy of the EVs in AS. Platelet mimetic‐EVs (P‐EVs) pass through the endothelium into plaques after being injected into mice with AS. They are substantially absorbed by inflammatory macrophages transmitting miRNAs into their cytoplasm. The miRNAs from these P‐EVs reduce the development of AS and stabilize the plaques by reducing the oxidative stress and inflammation and preventing the formation of foam cells.

#### 4.3.3 Cells Modified with Targeting Ligands

Cell signal transduction pathways, which are involved in biological processes, are characterized by various interactions between biomolecules, including reciprocal recognition between receptors and ligands.^[^ ^21^ ^]^ The accumulation and cellular uptake of NPs in atherosclerotic plaques can be improved by developing interaction‐based strategies. A protein called annexin V (AnxV) can specifically recognize phosphatidylserine, which is increased in atherosclerotic plaques. Gong et al. modified the surface of nano‐sponge‐like liposomes (Rb1‐LPs) with AnxV using a click reaction to obtain the final AnxV‐Rb1‐LPs product.^[^ ^108^ ^]^ AnxV‐Rb1‐LPs effectively accumulated in atherosclerotic plaques due to their passive and active targeting effects. Subsequently, they promoted atherosclerotic plaque regression by eliminating intracellular and extracellular cholesterol crystals. In addition, apoptotic cells express the “eat me” signal phosphatidylserine (PS), which has a strong affinity for SR‐BI and CD36 receptors on macrophages and causes them to be engulfed.^[^ ^122^ ^]^ To imitate the “eat me” signal and encourage cell absorption, Wu et al. employed PS‐coated nanomotors (using the high levels of ROS and the high level of expression of iNOS as the first step of targeting).^[^ ^123^ ^]^ The introduction of carrier‐free nanomotors has significantly enhanced the biological availability of trehalose, according to in vitro and in vivo data (the dose could be reduced from 2.5 g kg^−1^ in previous reports to 0.01 g kg^−1^ in this work). Furthermore, the consumption of ROS and production of NO during the targeting process both have beneficial functions; the former controls the M2 polarization of macrophages, whereas the latter encourages the restoration of the endothelial barrier, which ensures multitargeted treatment of AS.

### 4.4 Inorganic‐Based Nanomaterials

In recent years, nanomaterials have attracted increasing attention for targeted drug delivery and disease treatment in atherosclerosis due to their unique optical/ultrasound signal response properties.

Selective release of therapeutics for AS treatment from drug delivery systems in response to various remote applications and local stimuli, such as light, magnetic field, US, and pH, can be achieved by exploiting the unique magnetic, optical and/or physicochemical properties of metallo‐inorganic nanomaterials. Furthermore, therapeutics can be physically loaded into hollow or mesoporous inorganic nanostructures or chemically bound to nanomaterial surfaces for targeted drug delivery.

#### 4.4.1 Metal‐Based Nanomaterials

Metal‐based nanomaterials are widely used for imaging detection and medical evaluation of AS due to their unique metal responsiveness. Gold and iron‐based nanomaterials are commonly used for AS imaging diagnostics, specific response drugs, and stem cell delivery. Li et al. created a SPECT/CT imaging probe targeting apoptotic macrophages by coating gold nanoparticles (GNPs) with a thin layer of amino‐PEG and simultaneously coupling them with the targeting molecule membrane linked protein V and the radionuclide Tc‐99m.^[^ ^124^ ^]^ This maintains and enhances the targeting ability of membrane linked protein V to apoptotic macrophages. In the practical assessments, the intensity of the SPECT image correlated closely with pathological changes, while CT helped to better delineate the lesion. This imaging system thus helps to improve the localization and diagnosis of vulnerable Alzheimer's plaques by specifically targeting the apoptotic macrophages.

Mu et al. prepared ultrasmall dopamine‐modified hyaluronic acid (HD)‐stabilized Fe(III)‐tannic acid nanoparticles (HFTNPs).^[^ ^125^ ^]^ HFTNPs can specifically accumulate in inflammatory macrophages in atherosclerotic plaques, brighten MRI images, promote reactive oxygen species (ROS) generation, and induce inflammatory macrophage death without damaging normal cells and tissues. In addition, to investigate the imaging effect on the early lesions of atherosclerosis (foam cells), biomimetic Fe_3_O_4_ nanoparticles coated with a macrophage membrane (Fe_3_O_4_@M) were prepared. The Fe_3_O_4_@M effectively targeted early atherosclerotic lesions through specific recognition of integrin α4β1 to VCAM‐1, making it a promising contrast agent for early‐stage atherosclerosis diagnosis.^[^ ^126^ ^]^

#### 4.4.2 Composite‐Based Nanomaterials

Inorganic non‐metallic materials, such as silica and carbon nanomaterials, have received increasing attention in targeted drug delivery and disease treatment due to their porous structures and large surface areas. Pham et al. successfully produced CD9 antibody modified nanoparticles, CD9‐HMSN@RSV. These particles can carry a large amount of the anti‐aging drug RSV and release it via an HAase reaction, but also accurately target the ageing plaque of ApoE ‐/‐ mice and attenuate the ageing cells associated with atherosclerosis.^[^ ^127^ ^]^

In addition, there is an increasing interest in introducing inorganic nanomaterials into lipid or polymeric nanomaterials for the preparation of multifunctional NDDS containing contrast agents and therapeutic drugs, as well as the modification of inorganic nanomaterials using organic materials to improve chemical and physical properties, using multifunctional composites is of great importance to the treatment of AS.

For example, to create a multifunctional therapeutic nanoplatform, CS‐CNCs@Ce6/DS, Liu et al. prepared nanocarriers with chitosan‐coated carbon nanocages (CS‐CNCs) to load chlorin e6 (Ce6) and then attached dextran sulfate (DS) to the outermost layer by electrostatic adsorption. The nanoplatform selectively targets and accumulates on these macrophages, and DS can competitively inhibit the cellular endocytosis of oxidized low‐density lipoproteins by blocking the SR‐A receptor. Intriguingly, near‐infrared accelerated drug release was observed by the initial 808 nm laser exposure, increasing the Ce6 concentration in the plaque area and the efficiency of the photodynamic therapy.^[^ ^128^ ^]^ Kim et al. prepared silica‐coated iron oxide nanoparticles (SIONS) coated with rhodamine‐B isothiocyanate and polyethylene glycol, and investigated their feasibility in trapping macrophages in inflammatory plaques in apolipoprotein E‐deficient (*ApoE^−/−^*) mice. Following intravenous injection of SIONS, MR and FR imaging of the aorta was performed, and nanoparticle imaging increased the ability to monitor and treat *ApoE^−/−^* after treatment.^[^ ^129^ ^]^

In conclusion, the unique properties of inorganic nanomaterials can be used to prepare versatile and selective drug delivery or release systems for the treatment of AS and improve diagnostic tools.

## 5 Therapeutic Strategies for AS

### 5.1 Strategies based on Responses to the Atherosclerotic Microenvironment

In the past decade, stimulus‐responsive drug delivery systems have been increasingly recognized as bioinspired and powerful drug delivery platforms for the treatment of AS. Drugs loaded into stimulus‐responsive delivery systems that release medications in a specific affected area can improve the overall safety, delivery, and effectiveness of AS therapies. Drug localization and release at specific places can be accomplished via the stimulus response system using stimuli of a certain pathophysiological environment such as ROS, shear stress, pH, and enzymes. Here, we summarize stimulus‐responsive nanodrug delivery systems using for the treatment of AS (**Table** **1**).

**Table 1:** Responsive therapy strategies for the effective treatment of atherosclerosis.

Targeting strategy	Functional moieties	Delivery agents	Nanotechnology	Animal model	Therapeutic effects	Reference
pH‐responsive	ZIF‐8	Simvastatine (SIM)	SIM/ZIF‐8@HA	*ApoE^−/‐^* mice fed a high‐fat diet (HFD)	atherosclerotic lesions↓; lipid deposition↓	[79]
	Acid‐cleavable hydrazone bond	all‐trans retinal (ATR); rapamycin (RAP)	HR_RAP_	*ApoE^−/‐^* mice and fed a high‐fat diet (HFD)	atherosclerotic lesions↓; collagen content ↑; MMP‐9 level↓; intimal SMCs↓	[130]
ROS‐responsive	6s‐PLGA‐DAr‐PO‐PEG	probucol (PU)	RPP‐PU	*ApoE^−/‐^* mice and fed a high‐fat diet (HFD)	Visceral fat↓; abdominal aortic plaques↓;the area of plaques↓; the body fat rates of mice↓; the ROS levels in the aorta↓; collagen↓; TC, TG, LDL level ↓; the enzymatic activities of HMG‐CoA, LCAT, CETP, and APOE↓;	[131]
	CeO_2_		HA‐CeO_2_	*ApoE^−/−^* mice and fed a high‐fat diet (HFD)	the plaque areas of aortas↓;LDL level↓	[15]
	PMEMA	Prednisolone (Pred)	LFP/PCDPD	*ApoE^−/−^* mice and fed a high‐fat diet (HFD)	the area of plaques↓; the level of TNF‐α, MCP‐1 and IL‐1β↓; the level of H_2_O_2_ and Ox‐LDL↓	[132]
Shear stress‐Responsive	PEGDA	heparin	PEGDA nanogels	‐	thrombolysis	[133]
	PAMAM	synthesized simvastatin acid (SA)	SA PAM@RBCs	*ApoE^−/−^* mice and fed a high‐fat diet (HFD); New Zealand white rabbit with 30% FeCl_3_ to form thrombus	the blockage levels of rabbit carotid artery↓; the ROS level↓	[134]
Enzyme‐Responsive	HPGGPQ	RAP	RAP@T/R NPs	*ApoE^−/−^* mice and fed a high‐fat diet (HFD)	the area of plaques↓; the deposition of lipids↓; the rate of vascular stenosis↓; the number of macrophages↓; the expression of MMP‐9 and CTSK↓	[135]
	HA	RSV	CD9‐HMSN@RSV	*ApoE^−/−^* mice and fed a high‐fat diet (HFD)	The total cholesterol level↓; the level of SA‐β‐gal↓; the area of plaques↓;	[127]

#### 5.1.1 pH‐Responsive Strategies

**Figure 2:**
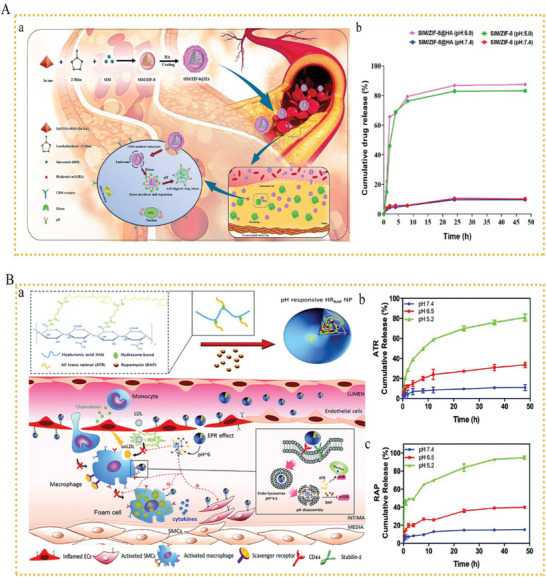
A) a) Schematic representation of the pH‐responsive SIM/ZIF‐8@HA. b) Drug release curve for SIM from SIM/ZIF‐8 and SIM/ZIF‐8@HA at different pH values. Reproduced with permission.^[^ ^79^ ^]^ Copyright 2022, Royal Society of Chemistry. B) a) Schematic representation of the pH‐responsive HR_RAP_ NPs that release ATR and RAP at the intima and intracellular spaces. The release behaviors of b) RAP and c) ATR from HR_RAP_ NPs at different pH values. Reproduced with permission.^[^ ^130^ ^]^ Copyright 2022, Royal Society of Chemistry.

In the atherosclerotic lesion microenvironment, when the levels of macrophage increase, lactic acid levels increase, and the pH decreases from 6.8 to 5.5.^[^ ^136^ ^]^ Based on this low pH, Essam et al. used a one‐step self‐assembly method to effectively encapsulate the hypolipemiant SIM in ZIF‐8.^[^ ^79^ ^]^ Based on the pH‐sensitive properties of ZIF‐8‐derived NPs, SIM/ZIF‐8 was degraded in the atherosclerotic microenvironment ensuring a controlled release of the loaded drugs. After incubation for 48 h at pH 5, 87.5% of the SIM was released from SIM/ZIF‐8 due to the disruption of the coordination bonds between the zinc ions and ZIF‐8′s 2‐methylimidazole in an acidic environment. However, there was no discernible release of SIM from the NPs after 48 h of incubation in a neutral solution. Cheraga et al. constructed pH‐sensitive nanoparticles (HR_RAP_ NPs) to co‐deliver all‐trans retinal (ATR) and rapamycin (RAP).^[^ ^130^ ^]^ HA was connected to antioxidants using a pH‐sensitive hydrazone bond and further loaded with RAP. After 48 h and at pH 7.4, the release rate of ATR was < 10%. However, over the 48 h period, the cumulative release at lower pH levels rose dramatically, reaching ≈33.5% at pH 6.5 and 80.3% at pH 5.2. Similar to ATR, RAP was released at a slower rate at pH 7.4; however, it was rapidly released at a rate of 39.8% and 94.9% within 48 h at pH 6.5 and 5.2, respectively. The association of ATR‐RAP with HR_RAP_ NPs may have synergistic effects as the anti‐inflammatory properties of RAP and the antioxidant activity of ATR in vitro may simultaneously reduce ROS levels and inflammation. The association ATR‐RAP in HR_RAP_ NPs may have synergistic effects as the anti‐inflammatory properties of RAP and the antioxidant activity of ATR may simultaneously reduce ROS levels and inflammation. These results show that it is possible to increase drug bioavailability and drug synergism using prodrugs and drug envelopes. Designing a medical delivery system to target atherosclerotic lesions with low‐pH environments is clearly a promising strategy (**Figure** **2**).

#### 5.1.2 ROS‐Responsive Strategies

In AS, ROS may accumulate as a result of persistent inflammatory activity in localized affected regions.^[^ ^137^ ^]^ ROS consist of oxygen free radicals and peroxides that determine macrophage infiltration and ECs damage.^[^ ^138^ ^]^ Thus, utilizing antioxidant drugs in delivery systems made by materials with inherent ROS‐scavenging properties may be an effective and promising treatment strategy for atherosclerotic lesions. Liang et al. designed a new H_2_O_2_‐scavenging polymer, polylactic glycolic acid‐diphenyl ring‐peroxyoxalate bond‐polyethylene glycol.^[^ ^131^ ^]^ The conjugation of the diphenyl ring with the peroxyoxalate bond increased the sensitivity to hydrogen peroxide; the desired reaction could be obtained at 10 µmol L^−1^, which is close to the lowest concentration of H_2_O_2_ in the pathological environment. At the same time, a H_2_O_2_‐scavenging nano‐bionic system decreased ROS to normal levels in lipopolysaccharide‐stimulated RAW and endothelial cells in vitro. These NPs dramatically decreased the body fat percentage, serum levels of LDL, vascular plaque area, and the development of fibrous tissue and collagen in atherosclerotic lesions in *ApoE^−/−^* mice. Cerium oxide nanozymes are currently receiving considerable attention because of their superoxide dismutase and catalase‐mimicking activities. Wang et al. used HA‐guided nanozyme technology and discovered that HA‐CeO_2_ NPs efficiently prevented oxLDL endocytosis by activated macrophages.^[^ ^15^ ^]^ These NPs actively target plaque‐associated macrophages eliminating excessive ROS and protecting macrophages from ROS‐induced damage.

**Figure 3:**
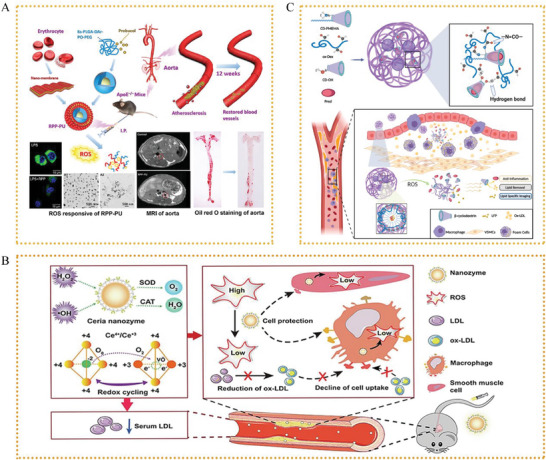
Structural illustrations showing A) the therapeutic mechanism of RPP‐PU. Reproduced with permission.^[^ ^131^ ^]^ Copyright 2022, Elsevier Ltd. B) The therapeutic mechanism of HA‐CeO_2_ NPs. Reproduced with permission.^[^ ^15^ ^]^ Copyright 2022, Elsevier Ltd. C) The therapeutic mechanism of LFP/PCDPD, reproduced with permission.^[^ ^132^ ^]^ Copyright 2022, Elsevier Ltd.

To achieve the accurate and efficient release of anti‐inflammatory medications from nanocarriers in atherosclerotic areas, high levels of ROS can be used as a trigger. Therefore, developing a nanomedical drug delivery system with ROS‐reactive drug release will ensure both an active targeting of atherosclerotic lesions and considerable therapeutic effects of drugs against AS. Xu et al. constructed dextran‐based ROS‐responsive NPs (LFP/PCDPD) loaded with a lipid‐specific aggregation‐induced emission fluorescent probe (LFP).^[^ ^132^ ^]^ In the presence of ROS‐rich microenvironments in AS, poly(2‐(methylthio)ethyl methacrylate) changes from hydrophobic to hydrophilic, resulting in drug release. Additionally, cyclodextrin can release the loaded drug with the occurrence of the therapeutic effects as well as ensure lipid clearance. LFP/PCDPD NPs have demonstrated superior therapeutic performance in both in vivo and in vitro evaluations of anti‐AS treatments owing to this therapeutic approach.(**Figure** **3**).

#### 5.1.3 Shear Stress‐Responsive Strategies

**Figure 4:**
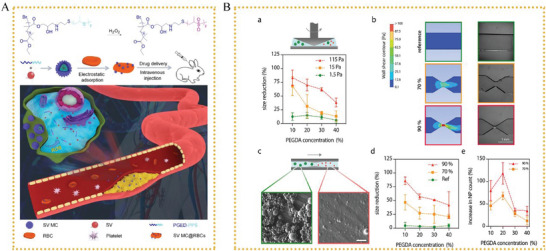
A) Synthesis process of SA PAM@RBCs. Reproduced with permission.^[^ ^134^ ^]^ Copyright 2021, Royal Society of Chemistry. B) Nanogel behavior under different mechanical loads. Reproduced with permission.^[^ ^133^ ^]^ Copyright 2021, Royal Society of Chemistry.

The shear stress levels in human arteries typically range from 1–6 Pa when in good health. However, AS‐induced pathological stenosis can result in excessively high wall shear stress levels of > 100 Pa.^[^ ^139^ ^]^ Therefore, mechanically sensitive drug delivery systems could be promising platforms for customized solutions designed to improve drug efficacy. Kimna et al. demonstrated the sensitivity of nanogels containing different poly(ethylene glycol) diacrylate concentrations to different shear stress levels.^[^ ^133^ ^]^ Similarly, simvastatin acid (SA)‐loaded cross‐linked dendrimer nanoparticles (SA PAM) were created by Shen et al. and attached to the surfaces of RBCs resulting in the formation of SA PAM@RBCs.^[^ ^134^ ^]^ While 61.3% of the SA PAM desorbed from RBCs in response to a high shear stress stimulus (100 dynes per cm^2^), only 12.4% detached from RBCs under low shear stress (20 dynes per cm^2^). This strategy could effectively suppress ROS levels in LPS‐induced RAW 264.7 cells and boost medication accumulation in plaques to enhance the therapeutic impact of SA and reduce the side effects caused by soluble SIM. In summary, drug delivery via shear stress‐sensitive release is a promising strategy for the treatment of AS.(**Figure** **4**).

#### 5.1.4 Enzyme‐Responsive Strategies

Increased levels of certain enzymes, such as hyaluronidase,^[^ ^140^ ^]^ matrix metalloproteinases (MMPs),^[^ ^141^ ^]^ and cathepsin K (CTSK),^[^ ^142^ ^]^ have been linked to AS and cardiovascular diseases. Increased enzyme levels may be suitable triggers for drug release from nanocarriers in atherosclerotic lesions. Fang et al. designed CTSK‐sensitive NPs to treat AS.^[^ ^135^ ^]^ The peptide sequence HPGGPQ (Dabcyl‐Lys‐HPGGPQ‐Glu [EDANS]‐acp‐Cys‐NH_2_) of type I collagen was created and synthesized to serve as the hydrolysis substrate for CTSK. Targeted and responsive NPs obtained using this CTSK‐sensitive polymer (PLGA‐Pep‐PEG) were obtained. The release of the active substance within this NPs was evaluated using a fluorescence resonance energy transfer probe. After the PLGA‐Pep‐PEG‐derived NPs were incubated with CTSK 10 nM for 8 h, the fluorescence intensity was 5.12 ± 0.13 and 4.50 ± 0.10 times greater than that of the NPs and PLGA‐Pep‐PEG without the CTSK treatment, respectively. Additionally, when the CTSK antibody was added to the PLGA‐Pep‐PEG polymer or NPs group, the fluorescence absorbance intensity was considerably reduced. NPs also enhanced RAP release in response to CTSK stimulation in vitro, significantly inhibiting OxLDL phagocytosis and cytokine release by pro‐inflammatory macrophages. These results show that the strategy based on enzyme‐sensitivity could be a potential treatment method for AS. Le Minh et al. developed a drug delivery platform that responded to hyaluronidase (HAase) using HA‐coated mesoporous silica‐based NPs.^[^ ^127^ ^]^ HAase (150 U mL^−1^) was used in two buffers to study the release of rosuvastatin (RSV). Compared to an environment lacking HAase, the environment containing HAase considerably accelerated RSV release at both pH levels, as the release rate reached 82.6% at pH 5.0 and 45.1% at pH 7.4. This quick rupture of the outer HA layer leads to accelerated buffer penetration into the mesoporous silica NP core, increased drug leakage, and may be the cause of the HAase‐responsive release of RSV.

Responsive therapy strategies have been suggested to improve drug‐targeting effectiveness, boost therapeutic benefits, and co‐delivery of different drugs. However, further research will be required to develop better theranostic drugs using combination therapies or agents that induce multiple responsiveness or possess increased sensitivity to stimuli at biologically meaningful doses.

### 5.2 Targeted Therapeutic Strategies for AS

Owing to their enhanced permeability and retention effect, nanodelivery systems have been widely used to deliver medicines specifically to atherosclerotic lesions while minimizing off‐target effects. However, the absence of site targeting is a drawback of conventional drug delivery nanosystems. The phagocyte system of the human body detects NPs as foreign materials resulting in their rapid clearance. This process is a key contributor to limited targeting. Therefore, while it is difficult, there is a critical need to engineer NPs that evade the phagocyte system but still achieve specific targeting (**Table** **2**).

**Table 2:** Targeting therapeutic strategies for the effective treatment of atherosclerosis.

Targeting strategy	Reception	Targeting ligands	Carriers	Delivery agents	Animal model	Therapeutic effects	Reference
Macrophage‐targeted		Macrophage membrane	PLGA nanoparticles	rapamycin (RAP)	*ApoE^−/−^* mice fed a high‐fat diet	lesion area↓; macrophage infiltration↓; collagen level; level of TNF‐α, IL‐1β↓; levels of LDL, triglyceride (TG), and total cholesterol (TC) ↓	[143]
		M2 macrophage membrane	Mixed membrane	simvastatin (SIM)	*ApoE^−/−^* mice were fed a high‐fat diet	lesion area↓; lipid deposition area↓; necrotic core areas↓; number of macrophages and SMCs↓	[107]
	CD44	HA	CeO2		*ApoE^−/−^* mice were fed a high‐fat diet	lesion area↓; plaque area↓; levels of LDL↓	[15]
		HA	Soybean phospholipid	Simvastatin (SIM)	*ApoE^−/−^* mice were fed a high‐fat diet	plaque size↓; lipid deposition↓; levels of IL‐6, TNF‐α and CCL2↓	[144]
	LXR receptor	D‐Nap‐GFFY	D‐Nap‐GFFY hydrogel	T0901317	*ApoE^−/−^* mice were fed a high‐fat diet	levels of ABCA1, ABCG1 and MerTK↓; macrophage population↓; levels of MMP‐9, MCP‐1 and CD68↓; levels of α‐SMA↑; plaque size↓	[75]
	Mannose receptors	Mannose	Se	Calpain inhibitory peptide, (CIP)	*ApoE^−/−^* mice were fed a high‐fat diet	lesion area↓; calpain activity; M2 macrophage↑; levels of IL‐6, TNF‐α↓; levels of IL‐10↑	[145]
	Type A scavenger receptor (SR‐A)	Dextran sulfate (DS)	Chitosan‐coated carbon nanocages	Chlorin e6 (Ce6)	*ApoE^−/−^* mice fed a high‐fat diet	plaque area↓; levels of IL‐1β, TNF‐α↓; levels of SR‐A	[128]
	Folate receptor(FR)	Folate	PLGA	Naringenin	*ApoE^−/−^* mice fed a high‐fat diet	lesion area↓; necrotic core area↓; levels of Glu, LDL, TC, TG↓; levels of HDL↑	[146]
	PS receptors (PSR)	PS		Trehalose (Tr)		endothelialization rate of the atherosclerotic aortas↑; continuity of CD31^+^ endothelial cells↑; lesion area↓; ratio of M2 macrophages↑; ratio of M1 macrophages↓; levels of TNF‐α and IFN‐γ↓; levels of IL‐10 and TGF‐β↑	[123]
	Stabilin‐2 receptors	S2P peptide	PLGA	siRNA	*ApoE^−/−^* mice fed a high‐fat diet	CaMKIIγ in lesional macrophages↓; efferocytosis↑;fibrous cap thickness↓; necrotic core areas↓	[147]
	Stabilin‐2 receptors	S2P peptide	PLGA	siRNA	myeloid‐specific‐deficient ABCG1 murine model	CD36‐mediated lipid uptake↓;ABCG1‐mediated cholesterol efflux↑; fibrous cap thickness↓; necrotic core areas↓	[148]
Inflammation‐Targeted	IL‐1 receptor	IL‐1 receptor antagonist	Copper‐doped mesoporous silica nanoparticles		*ApoE^−/−^* mice fed a high‐fat diet	plaque size↓; VCAM‐1 expression↓	[149]

#### 5.2.1 Macrophage‐Targeted Therapy

Given the significant role that macrophages play in the atherogenesis process, modulating their response is thought to be a promising approach for the treatment of AS. As a result, numerous NP platforms have been created, and great progress has been made in the targeting of plaque macrophages for the treatment of AS. The main objective of this section is to summarize existing work on the safe and efficient targeted medication delivery to plaque macrophages.

##### Macrophage Mimetics

**Figure 5:**
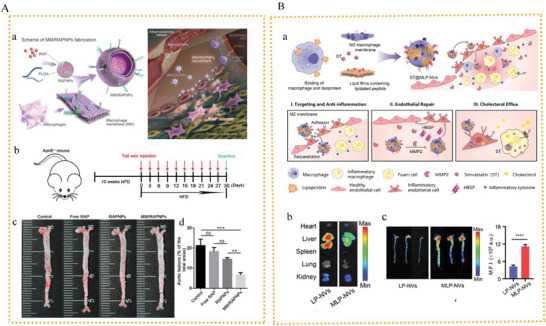
A) a) Schematic representation showing MM/DiDNPs.^[^ ^143^ ^]^ b) Establishment of experimental *ApoE^(‐/−^)* mice and administration c) Oil red O staining of lipid burden in aortas. d) Quantitative analysis of lipid burden in aortas. Reproduced with permission.^[^ ^143^ ^]^ Copyright 20221, Ivyspring International Publisher. B) a) Schematic representation showing MLP‐NVs.^[^ ^107^ ^]^ b) IVIS images of nanovesicles in major organs of atherosclerotic mice. c) Representative fluorescent images of the whole aorta. Reproduced with permission.^[107]^ Copyright 2022, Wiley‐VCH.

Recently, nanofilm coatings have become both popular and successful tools used in nanotechnologies.^[^ ^150^, ^151^, ^152^ ^]^ Previous studies on macrophage‐coated NPs against many inflammatory disorders, such as rheumatoid arthritis and cancer, have demonstrated excellent targeted delivery and good therapeutic efficacy.^[^ ^153^, ^154^ ^]^ Macrophage membrane‐camouflaged NPs may be a potent tool for the targeted therapy of AS. Wang et al. disguised PLGA‐based NPs loaded with RAP (RAP NPs) with membranes of the macrophages for targeted and effective therapy of AS based on macrophage “homing” into atherosclerotic lesions.^[^ ^143^ ^]^ The targeting ability of macrophage membranes/1,19‐dioctadecyl‐3,3,39,39‐tetramethylindodicarbocyanine perchlorate (MM/DiD) NPs towards atherosclerotic regions was assessed in an *ApoE^−/−^* mouse AS model. 24 h after the injection, ex vivo imaging clearly showed that the accumulation of MM/DiDNP was largely located in the plaque area and that the fluorescence intensity was much higher than that of DiDNP. The pharmacokinetic experiment showed that the fluorescence signal of the liver and kidney in the MM/DiDNP group was significantly weaker than that in the DiDNP group. In addition, the use of a multi‐characteristic bionic concept that is unrestricted by the origin or function of a cell enables flexible personalization to treat multiple pathogenic factors in the complex plaque environment. Thus, Guo et al.^[^ ^107^ ^]^ created biomimetic hybrid nanovesicles (MLP‐NVs) by fusing macrophage membranes with lipidated peptides (DOPE‐pp‐HBSP). 8 h after injecting the DiD‐labeled NVs, the major organs of the atherosclerotic and healthy mice were removed for ex vivo imaging. In the atherosclerotic mice, treatment with MLP‐NVs generated a lesser signal in the liver than that with LP‐NVs, and this was possibly because MLP‐NVs express the CD47 protein that decreases internalization via the reticuloendothelial system and increased blood circulation. 8 h after delivery, MLP‐NVs induced a clear fluorescence signal in the aortas, with 1.5‐ and 2.7‐fold higher intensities than M0‐NVs and LP‐NVs, respectively. Thus, MLP‐NVs improved the selective accumulation in plaques.(**Figure** **5**).

##### CD44

**Figure 6:**
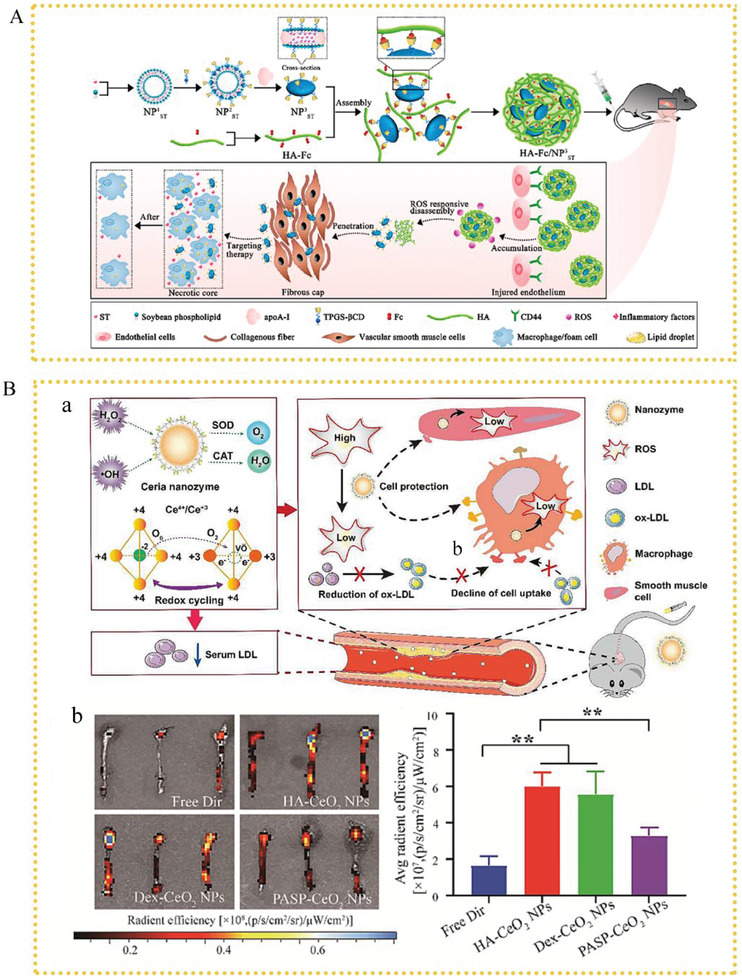
A) Schematic illustration showing the mechanism of HA‐Fc/NP3ST nanoassemblies. Reproduced with permission.^[^ ^156^ ^]^ Copyright 2023, Elsevier Ltd. B) a) Structure illustration showing the therapeutic mechanism of HA‐CeO_2_ NPs. b) Representative fluorescence images of the aorta and fluorescence intensity. Reproduced with permission.^[^ ^15^ ^]^ Copyright 2022, Elsevier Ltd.

CD44 is a multifunctional transmembrane glycoprotein that is overexpressed in various activated macrophages and serves as the major receptor for HA.^[^ ^155^ ^]^ Numerous studies have consequently used HA modifications for the distribution of medication preferentially into the atherosclerotic plaque, by targeting intraplaque macrophages. He et al. successfully developed ROS‐responsive size‐reducible hyaluronic acid‐ferrocene /β‐cyclodextrins‐anchored discoidal recombinant high‐density lipoprotein (HA‐Fc/NPs^3^) nanoassemblies for targeted AS therapy.^[^ ^156^ ^]^ Through the recognition of HA‐CD44 receptors, HA‐Fc/NP^3^ successfully traversed the damaged endothelium, disintegrated quickly in to the presence of ROS, entered into the macrophage spheroids, and facilitated macrophage‐targeted cholesterol efflux. Atherosclerotic plaque‐associated macrophages strongly colocalized with HA‐Fc/NP^3^_ST_, which preferentially accumulated in an area prone to AS. This was caused by the HA‐based formulation's extremely effective internalization, which was stimulated by CD44‐mediated endocytosis. To reduce the excessive formation of ROS in AS, Wang et al. created biocompatible HA‐guided assemblies of HA‐CeO_2_‐based NPs as plaque‐targeting ROS scavengers.^[^ ^15^ ^]^ As shown by in vivo imaging tests, HA‐CeO_2_ NPs had excellent plaque‐targeting effects because of the proinflammatory macrophage‐mediated rapid endocytosis. Fluorescence intensities in the aorta of mice treated with Dir‐labeled HA‐CeO_2_ NPs were approximately three times stronger than those of mice given free Dir.(**Figure** **6**).

##### Liver X Receptor

Liver X receptor (LXRs) are transcription factors that act as master regulators of lipid homeostasis as they function as “cholesterol sensors”, and their activation leads to a reduction in AS.^[^ ^157^ ^]^ Given the macrophage‐targeting ability of the naphthylacetic acid modified D‐glycine–phenylalanine–phenylalanine–tyrosine tetrapeptide (D‐Nap‐GFFY) and the key role of macrophages during AS development, Chuanrui et al.^[^ ^158^ ^]^ encapsulated T0901317 using a D‐Nap‐GFFY‐based hydrogel (named D‐Nap‐GFFY‐T0901317 or GFFY‐T0901317) to selectively target macrophages. D‐Nap‐GFFY‐T0901317 was ingested by macrophages including those that invaded the plaque or the liver through Kupffer cells, and it consequently blocked the foam cell generation and plaque inflammation caused by macrophages. The selective uptake of D‐Nap‐GFFY‐T0901317 by Kupffer cells in the liver can lead to the intracellular release of T0901317, indicating that T0901317 has anti‐inflammatory activity as it improved M2 polarization of the Kupffer cells. Additionally, the effect of the released T0901317 on hepatic lipogenesis was successfully blocked by the preferential uptake of D‐Nap‐GFFY‐T0901317 by Kupffer cells rather than hepatocytes.

##### Mannose Receptors

Mannose can specifically recognize the mannose receptor on macrophages and has frequently been used in nanovaccines.^[^ ^159^ ^]^ Yang et al. created a nanosystem using immune‐stimulating selenium in the form of calpain inhibitory peptide (CIP)‐loaded D‐mannose‐modified selenium‐based NPs (MSeNP@CIP).^[^ ^145^ ^]^ D‐mannose modification led to selective binding to mannose receptors and decreased calpain activity, boosting the accumulation of MSeNP@CIP in atherosclerotic plaques in ApoE^−/−^ mice with lower AS levels. Moreover, MSeNP@CIP demonstrated strong anti‐inflammatory effects via the regulation of the M1/M2 macrophage ratio. Together, these studies lay the groundwork for targeted nanotherapies that can reduce atherogenesis.

##### Type A Scavenger Receptor (SR‐A)

Class A scavenger receptors are overexpressed on the surfaces of activated macrophages that are early pathogenic alterations observed in AS.^[^ ^160^ ^]^ Only macrophages that have been activated overexpress SR‐A, and this mechanism can be utilized to target macrophages effectively. Dextran sulfate (DS), an SR‐A ligand, is a polyanionic derivative of dextran with a significant negative charge, that is safe, biocompatible, and biodegradable.^[^ ^161^ ^]^ Liu et al. created a chitosan‐coated carbon nanocage loaded with Chlorin e6 to target active macrophages using a DS ligand and performed sequential photothermal/photodynamic therapy.^[^ ^128^ ^]^ The release of Ce6 from nanocages can be accelerated by near‐infrared irradiation under an 808 nm laser. Ce6 produces singlet oxygen in the presence of a 633 nm laser that destroys macrophages and prevents the production of pro‐inflammatory cytokines and SMCs migration.

##### Folate Receptor (FR)

Active macrophages have been shown to dramatically overexpress FR‐beta near areas of inflammation; however, resting or quiescent macrophages express this receptor at relatively low levels.^[^ ^162^ ^]^ As a result, this characteristic has been used to deliver small‐molecule imaging agents and therapeutic conjugates specifically by folate molecules in a number of inflammatory disorders such as AS,^[^ ^163^ ^]^ osteoarthritis,^[^ ^164^ ^]^ rheumatoid arthritis,^[^ ^165^ ^]^ and systemic lupus erythematosus.^[^ ^166^ ^]^ These investigations have shown that folate‐targeted small‐molecule conjugates can efficiently aggregate at inflammatory sites, deliver medicines with precision, and treat illnesses. Guo et al. created folic acid‐adorned oral lipid‐polymer‐based nanoparticles (FA‐LNPs) to actively target aortic plaque sites and accumulate in lesion macrophages. Furthermore, the FA‐LNPs successfully overcame the intestinal mucosal‐epithelial barrier by increasing transmembrane transport through the intestinal epithelium.^[^ ^146^ ^]^ Using FA‐derived ligands, which specifically bind to overexpressed FRs on the surfaces of Ml macrophages in atherosclerotic plaques, nanomedicines can effectively penetrate the plaques of the atherosclerotic aortas of *ApoE^−/‐^* mice. Their oral administration determines the reduction of the aortic lesion area, plaque area, and necrotic core area of the aortic root in atherosclerotic *ApoE^−/‐^* mice.

##### PS Receptors

An anti‐inflammatory response is triggered and proinflammatory macrophages (M1) are induced into reparative phenotypes (M2) in response to macrophage endocytosis of apoptotic cells.^[^ ^167^ ^]^ The externalized PS on the membranes of apoptotic cells selectively binds to the PS receptors (PSRs) on the surfaces of macrophages, which accounts for endocytosis.^[^ ^168^ ^]^ Wu et al. used the “eat me” signal transmitted by PS to build nanomotors.^[^ ^123^ ^]^ The chemotactic behavior of the nanomotors is due to the specific affinity between iNOS at the AS site and an NO‐driven nanomotor that allows first‐step targeting to the AS site. To encourage macrophage cell uptake, PS‐coated nanomotors can mimic an “eat me” signal (second step targeting). The trehalose‐L‐arginine‐phosphatidylserine (TAP) nanomotor with the above two‐step targeting capabilities was 4.6 times more efficient than the TP NPs with one‐step targeting capabilities.

##### Stabilin‐2 Receptors

The first siRNA medicine to be available in the market, Onpattro (Patisiran) from Alnylam, received FDA approval on August 10, 2018, and subsequently, there has been a great amount of interest in RNA therapy. However, the inherent instability issues of naked RNA affect its use in the treatment of atherosclerosis. Lipid nanoparticles, however, have shown promise as a delivery system for RNA as they address the inherent stability problems with the naked RNA, increasing the therapeutic efficacy. For example, Tao et al. loaded siRNA to target the plaque‐destabilizing macrophage molecule Ca^2+^/calmodulin‐dependent protein kinase using S2P peptides connected to the lipid–polyethylene glycol layer on the surface of liposomes.^[^ ^147^ ^]^ The results showed that the knockdown of Ca^2+^/calmodulin‐dependent protein kinase in macrophages promoted efferocytosis, increased the thickness of the fiber cap and inhibited plaque necrosis to some extent. Similarly, Cui et al. used the S2P peptide to target diseased macrophages to deliver siRNA and found that the silencing of macrophage Epsins could reduce the size of atherosclerotic plaques and promote plaque regression.^[^ ^148^ ^]^ Mechanically, Epsins bind CD36 and promote lipid absorption by enhancing CD36 endocytosis and circulation. In contrast, Epsins promote ABCG1 degradation through lysosomes, block ABCG1‐mediated cholesterol effluence, and reverse cholesterol transport.

#### 5.2.2 Inflammation‐Targeted Therapy

The design of nanoplatforms for anti‐inflammatory therapy, diagnosis, monitoring, and immunological homeostasis balancing has increased exponentially owing to synergistic advancements in nanotechnology, biomaterials, and immunology.^[^ ^169^ ^]^ An inflammation‐specific nanoplatform is often built to respond to the inflammatory milieu after nanocarriers arrive at the target region. Designing nanoplatforms to evade systemic clearance by the RES, to enhance tissue biodistribution by focusing on inflammatory cell receptors, and to facilitate navigation to the inflammatory microenvironment has received a large amount of attention.

##### Receptor‐Targeted Therapy

IL‐1 is a potent proinflammatory cytokine produced during the progression of AS.^[^ ^170^ ^]^ The IL‐1 receptor antagonist (IL‐1Ra), an IL‐1 family member that functions as an endogenous competitive inhibitor of IL‐1, modifies the interactions between the cytokine and its receptors, as well as downstream signaling. Thus, IL‐1Ra inhibits macrophage inflammation by competitively binding to IL‐1R and preventing the activation of intracellular signaling.^[^ ^171^ ^]^ Wu et al. created IL‐1Ra‐loaded copper‐doped mesoporous silica NPs (IL‐1Ra@Cu‐MSNs), which is a nanoplatform designed to target inflammation, for the simultaneous delivery of IL‐1Ra and Cu ions.^[^ ^149^ ^]^ The main proinflammatory factors responsible for ongoing inflammation in atherosclerotic lesions are IL‐1, IL‐6, and TNF‐α. The serum and carotid plaque levels of these inflammatory factors were assessed using an enzyme‐linked immunosorbent assay after 4 weeks of modeling. The IL‐1Ra@Cu‐MSNs‐treated group was found to have significantly lower IL‐1, IL‐6, and TNF‐α serum levels than the control group, and the effects were more pronounced than those of the Cu‐MSNs‐ or IL‐1Ra‐treated groups. Several different ligands bind to the transmembrane receptor for advanced glycation end products (RAGE). When endothelial RAGE binds to its ligands, various signal transduction cascades are activated, increasing the production of transcription factors such NF‐κB, activating protein‐1 and signal transducers and activators of transcription 3 (STAT3). Besides upregulating RAGE expression, this results in the generation of proinflammatory cytokines, chemokines, and cell adhesion molecules, which encourages the adherence of circulating leukocytes and intensifies vascular inflammation.^[^ ^172^, ^173^ ^]^ To deliver short hairpin RNA sequences specifically targeting RAGE, Cristina et al. created P‐selectin‐targeted cationic PEGylated liposomes (Psel‐lipo/shRAGE lipoplexes).^[^ ^174^ ^]^ The intravenous administration of Psel‐lipo/shRAGE lipoplexes in *ApoE^−/−^* mice significantly decreased the NF‐kB (60%–70%, *p* < 0.05) protein level when compared to Scr‐lipo/shRAGE, Psel‐lipo/shCTR, or PBS.

##### Endothelium‐Targeted Therapy

Endothelial dysfunction is a precursor of AS.^[^ ^175^ ^]^ Owing to their overexpression and specificity, inflammatory markers of endothelial damage serve as targets for medication delivery mechanisms.^[^ ^176^ ^]^ Wu et al. loaded puerarin, an anti‐inflammatory drug in a peptidic drug delivery platform possessing both integrin targeting ability and matrix metalloproteinase‐directed self‐assembly.^[^ ^177^ ^]^ An animal model of AS was obtained using *ApoE^−/−^* mice through the partial ligation of the left carotid artery. Their results showed that plaques were significantly smaller in the group treated with the peptidic dual‐targeting drug delivery system when compared with the Puerarin and single targeted peptide groups. This demonstrates that targeting signaling molecules and proteins in ECs can enhance the therapeutic effects of puerarin. Activated vascular ECs release VCAM‐1, an adhesion molecule that draws inflammatory cells to the active endothelium surface, at the location of atherosclerotic lesions. As a result, nanocarriers can specifically target ECs in AS plaques by delivering peptides that are identified by VCAM‐1. Nicholas et al. modified the VHPK peptide on the surface of poly(β‐aminoester) to construct VHPK NPs that specifically targeted the aortic ECs of mice.^[^ ^178^ ^]^ Furthermore, intravenous injections of VHPK NPs in an AS mouse model showed a significant accumulation of the plasmid DNA carried by the NPs within the atherosclerotic plaque sites.

The pharmacokinetic characteristics and bioavailability of currently available cardiovascular medications have been greatly enhanced by the logical design and deployment of tailored technologies in drug delivery. For example, Gao et al. encapsulated hydrophobic components from natural plants (curcumin and tanshinone IIA) to produce peptide HDL (pHDL) nanoparticles. The results showed that free curcumin reached its maximum blood concentration in 1 h, while the pHDL nanoparticles reached their maximum concentration in 8 h, significantly extending the internal circulation time of the curcumin.^[^ ^179^ ^]^ Nanodelivery systems can also improve the biosafety of drugs. For example, Zheng et al. prepared a urease‐catalase, micromotor‐driven NE nanodrug delivery system by asymmetrically immobilizing the enzyme on the surface of the natural NE and then loading urokinase (UK) coupled silver (Ag) nanoparticles (Ag UK), and the results showed that nanodelivery systems could significantly reduce hemorrhagic side effects.^[^ ^180^ ^]^ These advantages of nanotechnologies have to some extent accelerated the clinical development of new drugs and improved the drawbacks of substances with serious side effects in traditional drug formulations or poor tolerance in physiological environments. However, difficulties such as manufacturing issues, elusive mechanisms, and high costs still exist for targeted NP systems. To ensure safety, researchers should also investigate the cyclic half‐life and long‐term toxicity of nanomaterials in the future.

## 6 Conclusions and Prospects

In recent decades, enormous efforts have been made to promote the translation of nanotechnologies into clinical practice. However, research on nanoparticles is still predominantly at the animal preclinical stage. To date, only a few nanomedicines have been approved for clinical use, and most of these have been for the diagnosis or treatment of tumors,^[^ ^181^ ^]^ although some have been used in the treatment of other hematological^[^ ^182^ ^]^ or chronic kidney diseases.^[^ ^183^ ^]^ While certain nanoparticles have performed well in animal studies, the differences between humans and animals makes their transition to clinical use difficult. In summary, while nanoparticle trials are currently showing excellent results, there is still a long way to go before they will be available clinically for the treatment of atherosclerosis.

Considering the severity and poor prognosis of AS, numerous nanomaterials have been investigated to improve current treatment strategies. Researchers have created nanosystems using two primary methods based on the pathogenesis of AS and the complexity of the microenvironment: 1) considering the intrinsic chemical activities of nanomaterials, such as ROS and lipid clearance; and 2) using nanomaterial‐based carriers to deliver active substances such as anti‐inflammatory or cholesterol‐lowering molecules as well as genes. These nanosystems can be constructed to perform multiple functions, including extending blood circulation and enhancing drug absorption, which could ultimately increase therapeutic efficiency. There is no denying that these strategies offer innovative ways to treat diseases. However, there is an imperative need for further extensive research to translate these strategies to a clinical setting. Future research should thus focus on 1) the administration mode, as currently, most administration is via intravenous injection, and more practical administration methods are required to increase clinical convenience; 2) bio‐safety, as it is unclear how nanomedicines work in vivo in terms of metabolism, distribution, biological effects, and mechanisms of action; 3) in vivo studies, as these should be transitioned gradually from *ApoE^−/−^* mice to large mammals; and 4) the method of preparation, as the preparation of nanosystems for AS frequently has low yields and is difficult to regulate. Overall, there is a need for simpler, more controlled, and reproducible nanosystems to facilitate clinical conversion.

Besides the challenges listed above, specific developments in AS nanomedicine are anticipated in the future. These include: 1) the simplification of NPs, as it will be necessary to thoroughly investigate their therapeutic impacts, minimize alterations without sacrificing targeting ability, and achieve a large degree of integration into diagnostics and therapeutic strategies; 2) the interactions between nanoparticles and pathological tissues will need to be fully elucidated, and studies in large animal models conducted; 3) most nanomedicines have only been phenotypically investigated (e.g., assessment of anti‐inflammatory and anti‐oxidative effects and their impact on the efflux of cholesterol), and research on the precise mechanisms (which include the exact areas of action and targets of molecular interactions) is required. In‐depth mechanistic investigations are expected to facilitate clinical use and the logical guidance of nanomaterial designs. 4) Medication bioavailability and drug delivery effectiveness should be enhanced to hasten clinical translation and clarify the mechanisms of the pharmacokinetics and potential long‐term negative effects of NPs.

In conclusion, the integration of numerous research approaches and novel advancements in AS treatment have been made possible by nanosystems. Although there are barriers preventing the practical implementation of AS nanomedicine, it is anticipated that as different disciplines advance, treatment systems based on NPs will usher in a new era in the management of AS.

## Conflict of Interest

The authors declare no conflict of interest.
